# X69R Is a Non-Essential Gene That, When Deleted from African Swine Fever, Does Not Affect Virulence in Swine

**DOI:** 10.3390/v12090918

**Published:** 2020-08-21

**Authors:** Elizabeth Ramirez-Medina, Elizabeth Vuono, Sarah Pruitt, Ayushi Rai, Ediane Silva, James Zhu, Lauro Velazquez-Salinas, Douglas P. Gladue, Manuel V. Borca

**Affiliations:** 1 Agricultural Research Service (ARS), Plum Island Animal Disease Center, Greenport, NY 11944, USA; Elizabeth.Ramirez@usda.gov (E.R.-M.); Elizabeth.Vuono@usda.gov (E.V.); Sarah.Pruitt@usda.gov (S.P.); Ayushi.Rai@usda.gov (A.R.); Ediane.Silva@usda.gov (E.S.); James.Zhu@usda.gov (J.Z.); Lauro.Velazquez@usda.gov (L.V.-S.); 2 Department of Pathobiology and Veterinary Science, University of Connecticut, Storrs, Mansfield, CT 06269, USA; 3 Oak Ridge Institute for Science and Education (ORISE), Oak Ridge, TN 37830, USA; 4 Department of Anatomy and Physiology, Kansas State University, Manhattan, KS 66506, USA

**Keywords:** ASF, ASFV, African swine fever

## Abstract

African swine fever virus (ASFV) is currently causing devastating outbreaks in Asia and Europe, and the ASFV strain Georgia (ASFV-G) is responsible for these outbreaks. ASFV-G is highly virulent and continues to be maintained in these outbreak areas, apparently without suffering significant genomic or phenotypic changes. When comparing the genome of ASFV-G to other isolates, a thus-far uncharacterized gene, X69R, is highly conserved and, interestingly, is similar to another ASFV uncharacterized gene, J64R. All sequenced ASFV isolates have one or both of these genes, X69R or J64R, suggesting that the presence of at least one of these genes may be necessary for ASFV replication and or virulence. The X69R gene is present in the ASFV-G genome while J64R is absent. To assess the importance of X69R in ASFV-G functionality, we developed a recombinant virus by deleting the X69R gene from the ASFV-G genome (ASFV-G-ΔX69R). ASFV-G-ΔX69R had the same replication kinetics in primary swine macrophage cultures as the parental ASFV-G, indicating that the X69R gene is not essential for ASFV-G viability or efficient replication in the main target cell during in vivo infection. In addition, swine intramuscularly inoculated with a low dose (10^2^ HAD_50_) of ASFV-G-ΔX69R developed a clinical disease indistinguishable from that induced by the same dose of the virulent parental ASFV-G isolate. Viremia values of ASFV-G-ΔX69R did not significantly differ from those detected in animals infected with parental virus. Therefore, deletion of the X69R gene from ASFV-G does not affect virus replication or virulence in swine.

## 1. Introduction

The virus family *Asfarviridae* contains only one member, African swine fever virus (ASFV), which is the etiological agent of African swine fever (ASF). ASFV has a large, double-stranded DNA genome of around 180–190 kilobases that encodes for over 150 open reading frames (ORFs). ASF has been endemic in several sub-Saharan African countries and Sardinia (Italy) for decades. Recently, epizootics of ASFV have occurred throughout parts of Europe and Asia, stemming from a single introduction of ASFV in the Republic of Georgia in 2007. Quickly, ASFV spread into the Caucasus region, and further into Armenia, Ukraine, Azerbaijan, and Russia. This spread continued, and now is causing outbreaks that have spread as far west as Poland, affecting both the domestic and wild pig populations [1]. In 2018, the virus was identified in China, and within only a few months every province of China was affected. In 2019, ASFV continued to spread into neighboring countries that included Vietnam, Cambodia, Mongolia, Laos, Timor Leste, Philippines, Indonesia, and Korea. In 2019, there was a single controlled outbreak of ASF in the Luxemburg region of Belgium, increasing the risk of continued spread throughout Europe. In early 2020, ASF was detected in Papua New Guinea, elevating concerns in Australia as the virus continues to infect naïve pig populations. All of these European and Asian outbreaks are due to a single ASFV strain that is highly contagious and lethal in domestic pigs. The rapid, seemingly uncontrollable spread of ASFV has the potential to cause significant death in commercial swine populations, resulting in substantial economic losses in the swine industry and worldwide protein availability shortages [2].

Currently, there is no commercial vaccine available to prevent ASF [1]. Development of experimental, live-attenuated ASF vaccines have relied on the production of recombinant field isolates by genetic manipulation, in which one or more genes were deleted from the field isolate [3,4,5,6,7,8,9,10,11,12]. Therefore, to understand the role of individual viral genes in virulence and how their possible manipulation could be used to develop experimental vaccines is of critical and urgent importance.

Whole genome sequencing has revealed that there are variations in genome size among different ASFV isolates. Like poxviruses, the diversity within the ASFV genome is localized primarily in the left and right terminal genomic regions [13,14]. It is predicted that ASFV forms head to tail catamers, similar to vaccinia virus, which could explain the exchange of nucleic acids in these regions [15]. ASFV variable regions comprise the left 35 kb and the right 15 kb ends of the genome, and deletions in these areas have occurred during adaptation of the virus in vitro [16]. These two regions contain the multigene families (MGFs), comprised of five different families of genes with similar sequence patterns. The multigene families of genes have similar sequence characteristics, and the number and sequence of the multifamily genes is variable in various genomes. In the left variable region, there are two orthologous genes that do not belong to any MGF, X69R, and J64R. However, both genes have been found singularly to be absent in some ASFV genomes, with one or the other gene remaining. Due to this observation, it is possible that X69R and J64R could overlap functionality. This, along with the fact that at least one copy of either X69R or J64R gene is present in almost all ASFV isolates, indicates that the function of X69R or J64R may be essential for virus replication or virulence. It has been reported that X69R facilitates virus replication in an in evaluated in an in vitro system [17]. Neither X69R nor J64R has been experimentally characterized in terms of their role in virulence in domestic swine.

Here we demonstrate that deletion of the X69R gene does not affect virus viability or replication in vitro. Importantly, deletion of the X69R gene from the genome of the ASFV Georgia2010 isolate (ASFV-G-ΔX69R) does not significantly alter virus replication or virulence in swine. ASFV-G-ΔX69R inoculated intramuscularly with 10^2^ HAD_50_ produced a clinical disease in domestic pigs indistinguishable from that induced by similar doses of the virulent parental ASFV-G isolate. In addition, viremia values during ASFV-G-ΔX69R infection do not drastically differ from those detected in animals infected with parental virus.

## 2. Materials and Methods

### 2.1. Cell Cultures and Viruses

Defibrinated swine blood was used for the isolation of primary swine macrophages, as previously described [18]. Mononuclear leukocytes were separated by flotation over a density gradient (specific gravity = 1.079). The monocyte/macrophage cell fraction was collected and cultured in Primaria flasks (Falcon, Franklin Lakes, NJ, USA) using standard macrophage media, as previously described, at 37 °C under 5% CO_2_. Adherent cells were detached from the plastic by using 10 mM EDTA in phosphate buffered saline (PBS), and were then reseeded into 6- or 96-well dishes at a density of 5 × 10^6^ cells per mL for use in the described assays.

ASFV Georgia (ASFV-G) field isolate was kindly provided from the Laboratory of the Ministry of Agriculture (LMA) in Tbilisi, Republic of Georgia by Dr. Nino Vepkhvadze [16].

Comparative growth curves between ASFV-G-ΔX69R and parental ASFV-G were performed in primary swine macrophage cell cultures. Preformed monolayers were prepared in 6-well plates and infected at an MOI of 0.01 (based on HAD_50_ previously determined in primary swine macrophage cell cultures). After 1 h of adsorption at 37 °C under 5% CO_2_, the inoculum was removed, and the cells were rinsed two times with macrophage media. Two mL of macrophage media was added and incubated for 2, 24, 48, 72, and 96 h at 37 °C under 5% CO_2_. At the indicated times post-infection, the cells were frozen at ≤−70 °C, and the thawed lysates were used to determine titers by HAD_50_/mL in the primary swine macrophage cell cultures. Virus titration was performed in 96-well plates of primary swine macrophages. The presence of virus was assessed by hemadsorption (HA), and virus titers were calculated as previously described [19]. 

### 2.2. Construction of the Recombinant Viruses

**Figure 1:**
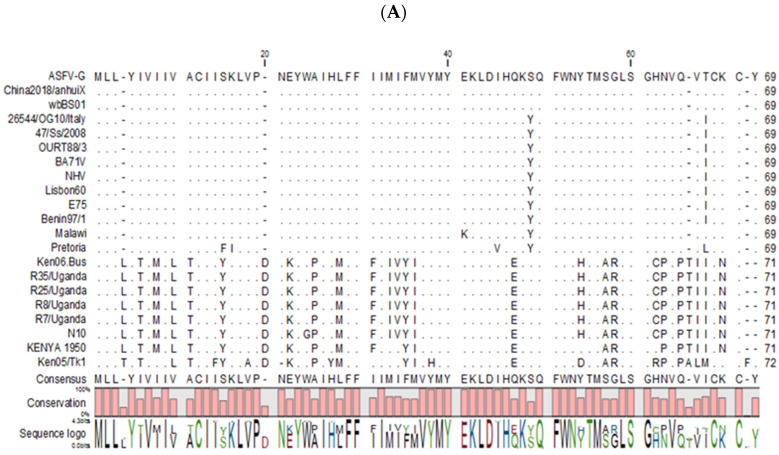

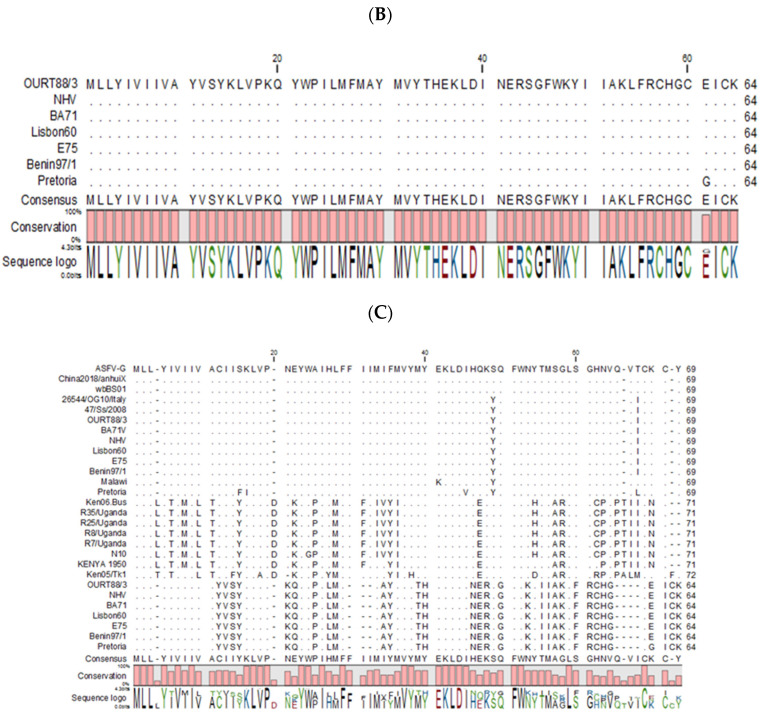
Multiple sequence alignment of the indicated African swine fever virus (ASFV) isolates for proteins. (**A**) X69R, (**B**) J64R, or (**C**) both X69R and J64R genes. Matching residues are represented as dots. The degree of conservation is presented below the protein sequence, and the conserved residue is presented on the bottom, indicating the degree of conservation for particular amino acids in the protein sequence.

Recombinant ASFV-G-ΔX69R was generated by homologous recombination between the parental ASFV genome and a recombination transfer vector (Figure 1) [18,20]. The recombinant transfer vector (p72mCherryΔx69R) contains a left recombination arm that is 1000 bp upstream of open reading frame (ORF) X69R, identical to ASFV-G nucleotide positions 18,227–19,226, followed by the previously described p72 promoter and mCherry Gene [21]; this is followed by a right recombination arm that is situated approximately 1000 bp downstream of X69R, identical to ASFV-G nucleotide positions 19,437–20,436. The recombinant transfer vector was obtained by DNA synthesis (Epoch Life Sciences, Sugar Land, TX, USA). Macrophage cell cultures were infected with ASFV-G and transfected with the transfer vector. Infection and transfection efficiency were evaluated by the visual observation of mCherry. The purification of the recombinant ASFV-G-ΔX69R was obtained by successive rounds of limiting dilution purification.

### 2.3. Microarray Analysis

The microarray data of ASFV ORFs were obtained from a previous study [22], where the data is available at the GEO repository under the series record GPL26,793. Background signal correction and data normalization of the microarray signals and statistical analysis were performed using the LIMMA package. The signal intensities of the ASFV ORF RNA were averaged from both Cy3 and Cy5 channels. The pattern of expression of the well-characterized ASFV early protein p30 (CP204L) and the late protein p72 (B646L) has been previously described, and was used here as a representation of early and late transcription profiles [15,16].

### 2.4. Complete Sequencing of ASFV Genomes Using Next-Generation Sequencing (NGS)

Macrophage cells were seeded as described and infected with ASFV; once the cytopathic effect was evident throughout the monolayer, DNA was isolated as described previously from cells infected with ASFV [21]. The extracted DNA was then used to completely sequence the virus DNA as previously described [21]. In brief, the viral DNA was sheared using enzymatic reactions assessed for the distribution of size fragmentation, then ligation of identifying barcodes using an adapter sequence was added to the DNA fragments. We then used this DNA library for next-generation sequencing (NGS) using the NextSeq (Illumina, San Diego, CA, United States). Sequence analysis was performed using CLC Genomics Workbench version 20 software (CLCBio, Waltham, MA, USA).

### 2.5. Animal Experiments 

Animal experiments were performed under biosafety level 3 conditions in the animal facilities at Plum Island Animal Disease Center (PIADC), following a strict protocol approved by the Institutional Animal Care and Use Committee (IACUC; 225.01-16-R_090716). ASFV-G-ΔX69R was assessed for its virulence relative to the parental ASFV-G virus, using 80–90 pound commercial breed swine. Five pigs were inoculated intramuscularly (IM) with 10^2^ HAD_50_ of ASFV-G-ΔX69R and compared with an additional group inoculated with similar dose of ASFV-G. Clinical signs (anorexia, depression, fever, purple skin discoloration, staggering gait, diarrhea, and cough) and changes in body temperature were recorded daily throughout the experiment. No particular scoring system was used, since clinical presentation of the disease in animals infected with the highly virulent ASFV strain Georgia can follow different patterns, from sudden death to a longer evolution, combining (although not obligatorily coexisting) different signs. IACUC protocol establishes specific points to proceed with humanitarian euthanasia after lack of mobility, severe anorexia, presence of neurological signs, or three consecutive days of very high body temperature.

## 3. Results

### 3.1. X69R Gene Is Conserved Across Different ASFV Isolates 

Sequence alignment from ASFV genomes that contain the X69R or J64R gene was performed. The multiple sequence alignment revealed a high degree of similarity among isolates. Results indicated that there are two main groups of ASFV isolates, based on the X69R gene sequence, with one group being similar to the current circulating strain of ASFV-G and another group obtained from sequenced isolates from Uganda and Kenya. When comparing the X69R sequence at the protein level between the two groups using ASFV-G and Kenya1950, X69R shared 69% nucleotide identity and 82% amino acid similarity (Figure 1A). It should be mentioned that genetic differences have been found among X69 genes from different isolates derived from the Georgia2007 isolates [23]. When comparing the multiple sequence alignment of the J64R translated product, this protein is almost 100% conserved among sequenced isolates (Figure 1B). When the two group alignments, X69R to that of J64R, were combined, the two proteins were 55% identical or 74% similar (Figure 1C), suggesting the possibility of a shared function between X69R and J64R, and possibly explaining why in some isolates only one of these proteins exist.

Of interest is the distribution of X69R and J64R in different ASFV isolates. Both genes are present in ASFV isolates Ba71V, Ourt88/3, NHV, Lisbon 60, E75, Benin97/1, and Pretoria/96/4. However, the X69R gene is only present in ASFV isolates Georgia2007/1, wb Bs01, China2018/anhuix, Malawi Lil-20/1, Kenya1950, R35/Uganda, R25/Uganda, R8/Uganda, and J64R R7/Uganda. The J64R gene is present in ASFV isolate Ba71, while X69R is absent. The alternative presence of orthologous X69R and J64R genes in several ASFV isolates suggests the possibility that these genes could have an overlapping, unknown function. Interestingly, there are no known ASFV isolates completely lacking the J64R and/or X69R genes, suggesting that at least one copy of the gene is necessary for virus replication in vitro or in vivo, or virulence in the natural host.

### 3.2. X69R Is Transcribed as an Early Viral Gene

**Figure 2:**
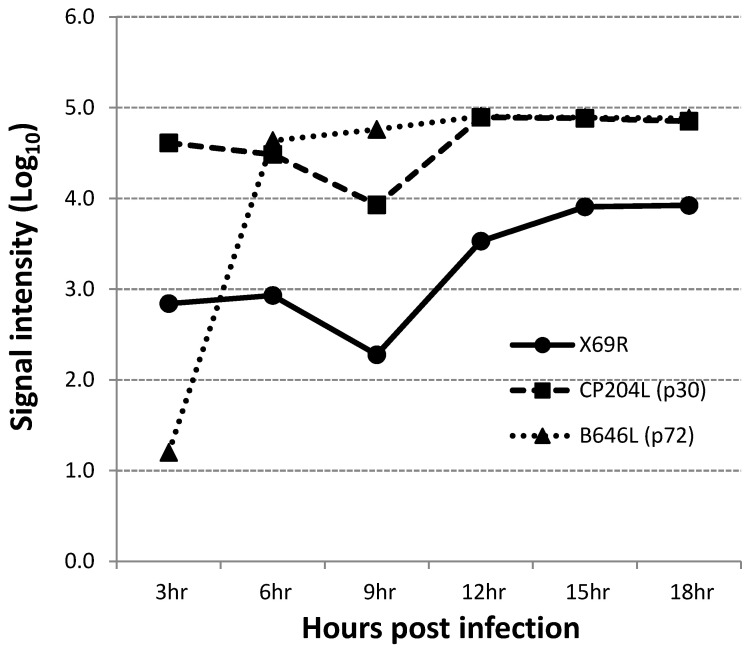
Time course of X69R gene transcriptional activity. Averaged microarray signal intensities (photons per pixel) of ASFV X69R, CP204L, and B646L open reading frame (ORF) RNA prepared from ex vivo pig macrophages infected with ASFV at 3, 6, 9, 12, 15, and 18 hpi.

To determine whether the X69R gene is actually transcribed during the infectious cycle, a time course experiment was performed to analyze the kinetics of RNA transcription in primary swine macrophages infected with ASFV strain Georgia. Swine macrophage cultures were infected with an MOI = 10 (to ensure the highest possible rate of cell infectivity in the cell culture); ASFV-G and cell lysate samples were taken at 3, 6, 9, 12, 15, and 18 hpi, completing approximately one virus replication cycle. The presence of X69R RNA was detected by DNA microarray analysis that was previously performed [22], as described in Material and Methods. Transcription of X69R was reliably detected at all time points, with signal-to-noise ratios (SNRs) of 18 or larger (an SNR of 3 is the threshold of reliable microarray detection); expression gradually decreased from 6 to 9 hpi, and then peaked at 12 hpi, followed by an increase from 12 to 18 hpi, like the trend observed during the period of 3 to 9 hpi (Figure 2). The pattern of expression of the well-characterized ASFV early protein p30 (CP204L) and the late protein p72 (B646L) has been previously described and is used here as a representation of early and late transcription profiles [6,24]. Therefore, the ASFV X69R gene encodes for a protein that is highly expressed early in the virus replication cycle.

### 3.3. Development of the ASFV-G-X69R Deletion Mutant

**Figure 3:**
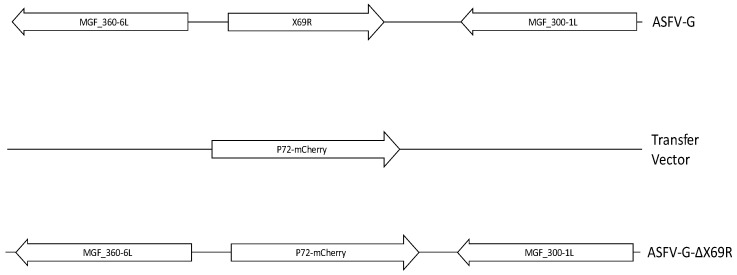
Schematic for the development of ASFV-G-ΔX69R. The transfer vector contains p72 promoter and the mCherry cassette, along with the flanking left and right arms of the transfer vector, designed to have flanking ends to both sides of the deletion/insertion cassette. The resulting ASFV-G-ΔX69R virus with the cassette inserted is shown on the bottom; the insert cassette is a direct replacement for the ORF X69R.

To determine the role of the ASFV X69R protein during ASFV infection in vitro and in vivo, a recombinant virus lacking the X69R gene was designed. Deletion of X69R was achieved by replacing the complete ORF with p72mCherry, following standard methodologies based on homologous recombination to generate recombinant ASFV viruses. The designed recombinant virus, ASFV-G-ΔX69R, was constructed from the highly pathogenic ASFV Georgia isolate (ASFV-G). A 403-bp region was deleted (between nucleotide positions 19,227–19,629) from the ASFV-G genome and replaced with a 1226 bp cassette containing p72mCherry (see Material and Methods) (Figure 3). The recombinant virus was obtained after nine successive limiting dilution purification events on monolayers of primary swine macrophage cell cultures. The virus population obtained from the last round of purification was amplified in primary swine macrophage cell cultures to obtain a virus stock.

To confirm that the only genetic modification was the deletion of X69R and that the integrity of the rest of the ASFV-G genome was maintained, full genome sequence of ASFV-G-ΔX69R was obtained by NGS on an Illumina NextSeq 500. The genome analysis confirmed the accuracy of the introduced modification and the absence of any additional significant mutations. In addition, NGS confirmed the absence of any residual X69R gene from parental ASFV-G genome as contaminant of the ASFV-G-ΔX69R stock.

### 3.4. Replication of ASFV-G-ΔX69R in Primary Swine Macrophages

**Figure 4:**
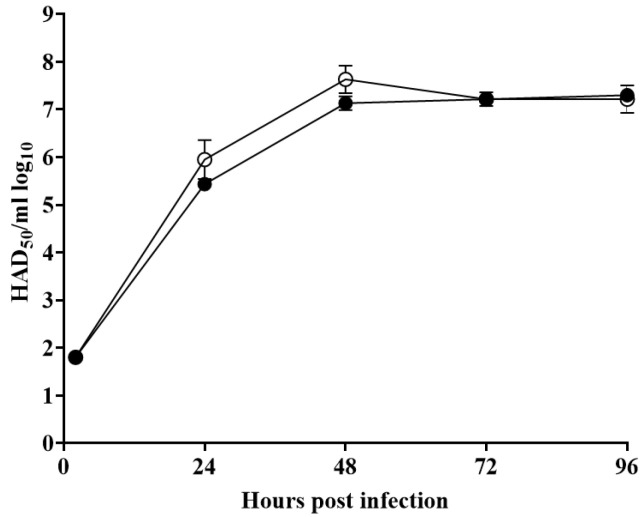
In vitro growth characteristics of ASFV-Georgia-ΔX69R (filled symbols) and parental (empty symbols) ASFV-Georgia (ASFV-G). Primary swine macrophage cell cultures were infected (MOI = 0.01) with each of the viruses, and virus yield was titrated at the indicated times post-infection. Data represent means and SD from three independent experiments. Sensitivity of virus detection: >1.8 log_10_ HAD_50_/mL.

To evaluate the role of the X69R gene, the in vitro growth characteristics of ASFV-G-ΔX69R were assessed in cell cultures of primary swine macrophages, the primary target cells during virus replication in swine, and compared them to parental ASFV-G in a multistep growth curve. The cell cultures were infected with these viruses at an MOI of 0.01, and samples were collected at 2, 24, 48, 72, and 96 hpi. A low MOI was used to ensure that the growth curve kinetics would cover as many replication cycles as possible, in order to increase the chance of detecting subtle differences in the comparative replicative abilities between ASFV-G-ΔX69R and the parental virus. Results demonstrated that ASFV-G-ΔX69R displayed an almost identical growth kinetic when compared to that of the parental ASFV-G virus (Figure 4). Therefore, deletion of the X69R gene does not significantly affect the ability of the virus to replicate in primary swine macrophage cultures.

### 3.5. Assessment of ASFV-G-ΔX69R Virulence in Swine

**Figure 5:**
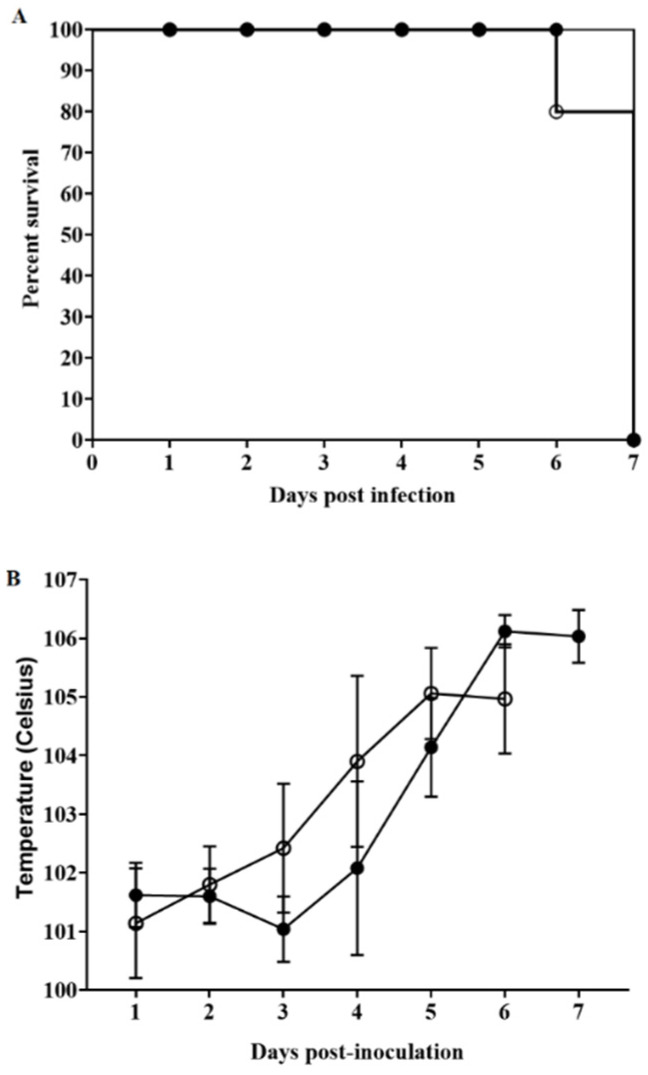
Progress of mortality (**A**) and body temperature (**B**) in animals intramuscularly (IM) infected with 10^2^ HAD_50_ of either ASFV-G-ΔX69R (filled symbols), or parental ASFV-G (open symbols). Panel (**B**) shows average data and the corresponding SD.

To evaluate the effect of the deletion of the X69R gene on ASFV-G virulence, groups of five 80–90 pound pigs were inoculated intramuscularly (IM) with 10^2^ HAD_50_ of either ASFV-G-ΔX69R or ASFV-G. Since ASFV Georgia is a highly virulent virus, with a very low 100% lethal dose, we decided to use a relatively low dose in order to increase the possibility of detecting subtle differences in virulence between ASFV-G-ΔX69R and the parental virus. A small reduction in virulence would be experimentally missed more easily if high doses were used. As expected, animals infected with ASFV-G exhibited an increased body temperature (>104 °F) by day 4 post-infection, followed by the appearance of clinical signs associated with the disease, including (although not obligatorily coexisting) high fever, anorexia, depression, purple skin discoloration, vomit, diarrhea, and neurological signs (Table 1 and Figure 5). Signs of the disease were aggravated progressively over time, and animals were euthanized in extremis by day 5–6 post-infection, following established regulations in the PIADC IACUC protocol. Interestingly, animals receiving 10^2^ HAD_50_ of ASFV-G-ΔX69R presented a disease evolution practically undistinguishable from those inoculated with ASFV-G: onset of the disease occurred by day 5 post-infection, and animals were severely sick and euthanized by day 6–7 post-infection. Both the time of presentation and severity of the clinical signs related with the disease completely resemble those present in animals inoculated with the parental virus. Therefore, deletion of the X69R gene does not significantly alter the virulence of the highly virulent ASFV-G isolate.

**Figure 6:**
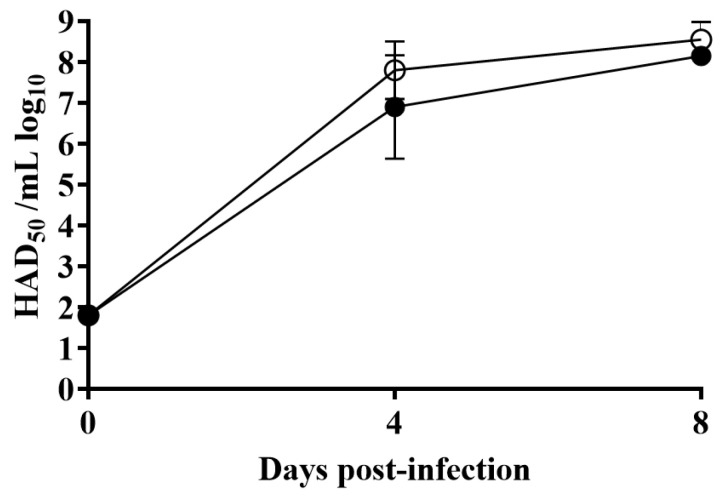
Viremia titers detected in pigs IM inoculated with 10^2^ HAD_50_ of either ASFV-G-ΔX69R (filled symbols) or ASFV-G (empty symbols). Each curve represents the average and SD of animal values in each of the groups. Sensitivity of virus detection: >log_10_ 1.8 HAD_50_/mL.

Analysis of viremia in animals infected with ASFV-G presented expected high titers (10^7^–10^8.5^ HAD_50_/mL) on day 4 post-infection, remaining high until day 7 post-infection, when all animals were euthanized. ASFV-G-ΔX69R-infected animals had viremias with values ranging from 10^4^ to 10^8^ HAD_50_/mL by day 4 post-infection, reaching titers similar to those of animals infected with ASFV-G by day 7 post-infection, which was the last sampling time before animals were humanely euthanized (Figure 6). Therefore, ASFV-G-ΔX69R virulence, in terms of clinical presentation and virological data, produces a disease indistinguishable from that induced by its highly virulent parental virus, ASFV-G.

## 4. Discussion

The majority of the 150 to 200 proteins encoded in the ASFV genome have not been experimentally characterized. Identifying viral proteins that are important for in vitro and in vivo virus replication, and importantly, in virus virulence in swine, is critical to developing novel countermeasures to control the disease. Discovery of ASFV gene function via genetic manipulation has enabled the production of experimental live-attenuated ASFV vaccine candidates by different research groups [3,4,5,6,7,8,9,25]. Interestingly, just a small number of virus genes have been successfully deleted from the ASFV genome, producing a novel recombinant virus (e.g., 9GL, UK, TK, MGF, NL, CD2, Lectin, DP148R, I177L, and C962R) [4,5,6,7,8,18,24,25,26,27,28,29,30,31,32]), and another small number of genes were determined to be essential for virus replication (e.g., EP152R, p30, p54, and p72) [27,33,34,35]. The absence of experimental information restricts the knowledge for most ASFV proteins to ORF analysis by functional genomics, predicting the functions of these ORFs.

In this study, we showed that X69R, a previously uncharacterized ASFV ORF, encodes a protein that is transiently expressed at early times during infection of swine primary macrophages, infected at a high MOI to ensure the synchronic infection of most of the cells in the culture. We also demonstrated that X69R is a non-essential gene, since its deletion from the ASFV-G genome does not significantly alter virus replication in swine macrophage cultures. These experiments were performed using a rather low MOI, with the purpose of ensuring that growth kinetics will require more than one replication cycle before the end of the experimental period. The additive effect of evaluating several successive replication cycles would enhance the possibility of detecting subtle differences between the replicative abilities of these viruses, which may not be appreciated in a single step growth curve like that performed at a high MOI.

Importantly, deletion of the X69R gene is not critical for ASFV virulence in swine, as the deletion mutant ASFV-G-ΔX69R had similar pathogenesis as the parental ASFV-G. Animals inoculated at very low doses (10^2^ HAD_50_) developed a disease indistinguishable from those receiving the parental, fully-virulent virus. The use of a relatively low dose increases chances of detecting small differences in virulence between ASFV-G-ΔX69R and ASFV-G, a virus with an unusually low 100% lethal dose.

We and others [35] have found sequence similarity between X69R and J64R genes, suggesting the possibility that these genes may play a similar function. Multiple findings support the potential critical role of these genes, including the fact that in all of the ASFV isolates, at least one of these genes is present (actually, both genes are present in a few other isolates), and interestingly, no sequenced isolate simultaneously lacking both of these genes has been identified. Therefore, the similarity between X69R and J64R proteins, and the fact that at least one of the genes is always present in the genome, suggests the possibility that one of these genes could be necessary for ASFV virulence, or at least for the process of virus replication. To our surprise, we were able to delete X69R and the resulting virus, ASFV-G-∆X69R, had similar replication rates both in vitro and in vivo, and presented an undistinguishable pathogenesis to that of the parental ASFV-G. In this regard, it is interesting to note that it has been reported that X69R protein, when individually expressed in ASFV-susceptible cells, increased the rate of virus replication [17].

It is interesting that neither X69R or J64R were detected in the ASFV proteome, [36] and that neither of them was detected as being part of the virus particle [37,38,39], obscuring the potential function of these genes. In addition, the molecular function of X69R or J64R has yet to be discovered. Further studies will have to be conducted to identify the exact molecular functions of either of these proteins, in order to determine the evolutionary role of why one of them has always been present in all field ASFV genomes evaluated so far (with no field isolates of ASFV lacking both the X69R and J64R protein). With the evolutionary advantage of viruses typically having a compacted genome, it is always surprising when the deletion of an ORF is non-essential, or at least does not affect to some degree the processes of both virus replication and virulence in the natural host. However, in the case of ASFV, and in particular with ASFV-G that exhibits experimentally 100% mortality at such a low viral dose, it is possible that the deletion of one gene may not drastically change the virulent phenotype. On the other hand, it is interesting to stress that there are situations where the deletion of one gene in ASFV is not possible, as some proteins have been shown to have essential functions. In addition, in rare cases, the deletion of one gene has shown to produce full attenuation of ASFV-G virulence in swine [5,6]. The lack of information of the essentiality of ASFV genes in either replication or in virus virulence is a significant gap in knowledge for basic ASFV virology, which requires further research to understand the necessary components to cause disease.

Therefore, these preliminary studies do not indicate that deletion of X69R in the context of the highly virulent Georgia2010 isolate affects virus replication or virulence in IM-infected swine. Further studies using a more natural route of infection (oronasal infection/cohabitation with infected donors), or by using a less virulent parental ASFV isolate will be required to entirely exclude a potential role of X69R in ASF pathogenesis. It is also possible that deletion of X69R gene may have a more pronounced phenotype in different hosts that are susceptible to ASFV infection, such as soft ticks or other Suidae, such as wild boar, and perhaps in one of these hosts a phenotype may be identified. Further studies in other hosts would be required to determine if this is the case. It is also possible that the function of X69R overlaps with other proteins in ASFV, and simultaneous deletion of these additional unknown proteins would be required in order to give a more pronounced phenotype and fully disclose the functional role of X69R. However, determining that the X69R gene can be deleted and showing that it is non-essential in virus replication and disease production is an important step for determining the potential minimal essential genome for ASFV. Improving our current understanding for the proteins required for the pathogenesis of ASFV and the viral molecular mechanisms that occur during infection can allow for the construction of better rational vaccine designs.

## Figures and Tables

**Table 1:** Swine survival and fever response following infection with ASFV-G-ΔX69R and parental ASFV-G.

Fever					
Virus (10^2^ HAD_50_)	No. of Survivors/Total	Mean Time to Death (±SD)	No. of Days to Onset (±SD)	Duration No. of Days (±SD)	Maximum Daily Temp., °F (±SD)
ASFV-G X69R	0/5	6.6 (0.55)	5.4 (0.55)	1.2 (0.45)	106.12 (0.28)
ASFV-G	0/5	5.6 (0.55)	4.2 (0.84)	1.2 (0.45)	105.6 (0.54)
